# Patient-Specific Finite Element Modeling of the Whole Lumbar Spine Using Clinical Routine Multi-Detector Computed Tomography (MDCT) Data—A Pilot Study

**DOI:** 10.3390/biomedicines10071567

**Published:** 2022-06-30

**Authors:** Nithin Manohar Rayudu, Karupppasamy Subburaj, Rajesh Elara Mohan, Nico Sollmann, Michael Dieckmeyer, Jan S. Kirschke, Thomas Baum

**Affiliations:** 1 Engineering Product Development (EPD) Pillar, Singapore University of Technology and Design (SUTD), Singapore 487372, Singapore; rayudu_nithin@mymail.sutd.edu.sg (N.M.R.); subburaj.karupppasamy@smu.ca (K.S.); rajeshelara@sutd.edu.sg (R.E.M.); 2 Sobey School of Business, Saint Mary’s University, 903 Robie St, Halifax, NS B3H 3C2, Canada; 3 Department of Diagnostic and Interventional Radiology, University Hospital Ulm, Albert-Einstein-Allee 23, 89081 Ulm, Germany; nico.sollmann@tum.de; 4 Department of Diagnostic and Interventional Neuroradiology, School of Medicine, Klinikum rechts der Isar, Technical University of Munich, Ismaninger Str. 22, 81675 Munich, Germany; michael.dieckmeyer@tum.de (M.D.); jan.kirschke@tum.de (J.S.K.); 5 TUM-Neuroimaging Center, Klinikum rechts der Isar, Technical University of Munich, 81675 Munich, Germany

**Keywords:** multi-detector computed tomography, finite element analysis, range of motion, spine, osteoporosis, vertebral fracture, bone mineral density

## Abstract

(1) Background: To study the feasibility of developing finite element (FE) models of the whole lumbar spine using clinical routine multi-detector computed tomography (MDCT) scans to predict failure load (FL) and range of motion (ROM) parameters. (2) Methods: MDCT scans of 12 subjects (6 healthy controls (HC), mean age ± standard deviation (SD): 62.16 ± 10.24 years, and 6 osteoporotic patients (OP), mean age ± SD: 65.83 ± 11.19 years) were included in the current study. Comprehensive FE models of the lumbar spine (5 vertebrae + 4 intervertebral discs (IVDs) + ligaments) were generated (L1–L5) and simulated. The coefficients of correlation (ρ) were calculated to investigate the relationship between FE-based FL and ROM parameters and bone mineral density (BMD) values of L1–L3 derived from MDCT (BMD_QCT-L1-3_). Finally, Mann–Whitney U tests were performed to analyze differences in FL and ROM parameters between HC and OP cohorts. (3) Results: Mean FE-based FL value of the HC cohort was significantly higher than that of the OP cohort (1471.50 ± 275.69 N (HC) vs. 763.33 ± 166.70 N (OP), *p* < 0.01). A strong correlation of 0.8 (*p* < 0.01) was observed between FE-based FL and BMD_QCT-L1-L3_ values. However, no significant differences were observed between ROM parameters of HC and OP cohorts (*p* = 0.69 for flexion; *p* = 0.69 for extension; *p* = 0.47 for lateral bending; *p* = 0.13 for twisting). In addition, no statistically significant correlations were observed between ROM parameters and BMD_QCT- L1-3_. (4) Conclusions: Clinical routine MDCT data can be used for patient-specific FE modeling of the whole lumbar spine. ROM parameters do not seem to be significantly altered between HC and OP. In contrast, FE-derived FL may help identify patients with increased osteoporotic fracture risk in the future.

## 1. Introduction

Metabolic bone disorders such as osteoporosis have become a prevalent medical condition among the elderly population worldwide [1]. Osteoporosis is a bone-related disorder associated with a reduction in bone mass and an increase in bone fragility [2,3]. Untreated osteoporosis can result in fragility fractures [1]. It is estimated that the prevalence of fragility fractures in the European Union will increase from 2.7 million in 2017 to 3.3 million by 2030 [4]. In addition, fracture-associated costs are estimated to grow 27% by 2030 from 37.5 billion in 2017 [4]. Besides hip fractures, vertebral fractures (VFs) are frequently reported osteoporotic fracture entities. They have been observed in around 30–50% of the population above 50 years of age [5]. The vertebral fracture occurrence can increase the subsequent vertebral fracture risk by 10-fold [6]. In addition, patients with a history of vertebral fractures have a 2.3-fold increase in hip fracture risk and a 1.4 times increase in distal forearm fracture risk [6]. Therefore, it is essential to identify subjects at risk for osteoporotic fractures early for better patient care.

The World Health Organization (WHO) recognizes a radiographic-based dual-energy X-ray absorptiometry (DXA) method for diagnosing osteoporosis [7]. The areal bone mineral density (aBMD) measures, i.e., T-scores and Z-scores derived from DXA, can be used to diagnose osteoporosis and estimate fracture risk [7,8]. However, studies have shown that the efficiency of the DXA-based method is limited (<50%) for predicting the fragility fracture risk [9]. Thus, the effectiveness of aBMD measures for osteoporotic vertebral fracture prediction is relatively low. Later, the University of Sheffield developed a statistical tool called the Fracture Risk Assessment Tool (FRAX) to assess the fracture risk [10]. FRAX calculates bone fracture risk using femoral neck BMD and 12 other parameters [11]. FRAX is an inexpensive, easily accessible web-based tool that does not require any technical expertise than the DXA-based method [12]. However, the effectiveness of the FRAX tool is limited due to relevant shortcomings [11]. Beyond DXA, quantitative computed tomography (QCT)-based volumetric BMD (vBMD) has been developed for quantitatively analyzing the bone with regard to osteoporosis [13,14]. This method provides more information as compared to DXA. However, studies have shown that the efficacies of aBMD-based and vBMD-based measures are limited in predicting osteoporotic fracture risk [9,15]. This is since BMD-based methods only consider the bone density values. However, other quantitative factors such as topology, size, bone mass distribution, and loading must be considered to understand and accurately quantify bone health.

Finite element (FE) patient-specific three-dimensional (3D) models derived from clinical multi-detector CT (MDCT) have been used widely for analyzing the bone qualitatively [16,17,18,19]. In this method, a patient-specific 3D anatomical model is segmented and reconstructed from the MDCT images. Then the model is meshed before applying image intensity-specific material properties and loading conditions. Next, boundary conditions are applied, and then the model is solved to derive structural and kinematic parameters. The MDCT-based FE method is widely used to evaluate vertebral failure load (FL), failure displacement, and range of motion (ROM) [15,17,20,21]. In addition, studies have shown that in vitro MDCT-based FE failure load values correlate well with the reported experiments and results in the literature [15,20].

Most previous studies have used CT-based images acquired in laboratory or research settings with higher resolution for building FE models of the spine than those acquired in the routine clinical settings for the diagnosis or monitoring [22,23,24,25]. The reported accuracies of these FE studies are promising. However, their clinical applicability is limited due to the need for high radiation doses during image acquisitions. Therefore, in recent studies, clinical routine MDCT data with lower spatial and in-plane resolutions were used for comparable FE analysis of individual vertebrae [17,26]. The lumbar spine is a complex anatomical structure comprised of different soft tissues, bones, and connecting elements (e.g., vertebrae, intervertebral disk (IVD), ligaments). These tissues constantly interact with each other and form an anatomically functional unit. Thus, it is essential to analyze the entire lumbar spine as a unit to better understand the spinal biomechanics and quantify fracture risk.

Thus, the current study aimed to investigate the feasibility of modeling and performing finite element simulation of the whole lumbar spine from routinely acquired in vivo clinical MDCT data for biomechanical analysis. To achieve the above-stated aim, we investigated the following objectives:

(1) Modeling and validation of the 3D patient-specific finite element model of the whole lumbar spine from routine clinical MDCT data (in vivo) for extracting biomechanical characteristics; and
(2) Comparison of these FE-derived structural biomarkers (FL and ROM) from whole lumbar spine models of healthy controls (HC) and osteoporotic patients (OP).

## 2. Materials and Methods

**Figure 1:**
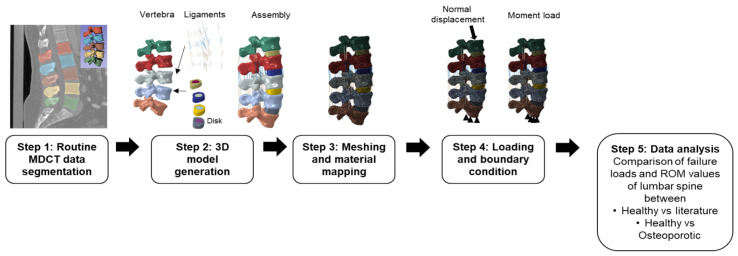
Schematic representation of the five-step modeling methodology used in this study for routine clinical MDCT image processing, modeling, and data analysis.

In the current computational study, we have followed a five-step methodology. In the first step, the routine MDCT data were automatically segmented. In the second step, the patient-specific 3D lumbar model was generated. Then the annulus fibrosus and nucleus pulposus of the intervertebral discs (IVDs) and ligaments were modeled separately and assembled with the vertebra. In the third step, the assembled lumbar model was meshed and image intensity-based material properties were mapped to the FE mesh. In step four, loading and boundary conditions were applied, and the model was solved. Finally, in step five, the data analysis was performed on the FE results. Figure 1 shows the schematic representation of the complete workflow followed in the current study.

### 2.1. Subjects

Twelve subjects, six HC (six females, mean age ± standard deviation (SD): 62.16 ± 10.24 years) and six OP (four males, two females, mean age ± SD: 65.83 ± 11.19 years), were included in this study. The digital picture archiving and communication system (PACS) of our institute was used to identify all included subjects retrospectively. 

All subjects with a known history of bone diseases, including metabolic, hematologic, and metastatic disorders other than osteoporosis, were excluded from the current study. The included subjects were categorized into healthy (QCT BMD > 80 mg/mL) and osteoporotic (QCT BMD < 80 mg/mL) subjects based on QCT BMD values derived from opportunistic use of clinical routine MDCT data [27,28] The QCT BMD values (BMD_QCT-L1-3_) for HC were as follows: 108.04, 107.82, 106.91, 103.86, 82.44, and 95.54 mg/mL, respectively. For OP, the BMD_QCT-L1-3_ values were as follows: 67.67, 62.44, 61.20, 57.14, 56.44, and 62.69 mg/mL, respectively. This retrospective study was approved by the local institutional review board (Faculty of Medicine, Technical University of Munich, ethics approval 2019, registration number: 27/19 S) and was conducted in accordance with the Declaration of Helsinki. The requirement for written informed consent was waived due to the study’s retrospective design.

### 2.2. Image Acquisition

A 64-row MDCT scanner (Somatom Sensation Cardiac 64; Siemens Medical Solutions, Erlangen, Germany) was used to acquire routine abdominal contrast-enhanced MDCT data according to clinical indications. A standard bone kernel was used to reconstruct the sagittal reformations of the spine with a 3 mm slice thickness. All image acquisitions were performed after administering an intravenous contrast medium (IVCM; Imeron 400; Bracco, Konstanz, Germany) using a high-pressure injector (Fresenius Pilot C; Fresenius Kabi, Bad Homburg, Germany). 

All scans were acquired using the following scanning parameters: minimum collimation of 0.6 mm, peak tube voltage of 120 kVp, average tube load of 200 mAs, and IVCM flow rate of 3 mL/s with a delay of 70 s. The amount of injected IVCM was based on the bodyweight of individual subjects: 80 mL for ≤80 kg, 90 mL for ≤100 kg, and 100 mL for >100 kg. In addition, all subjects received a 1000 mL oral contrast medium (Barilux Scan; Sanochemia Diagnostics, Neuss, Germany). For all acquired MDCT scans, a reference phantom (Osteo Phantom; Siemens Medical Solutions, Erlangen, Germany) was placed in the scanner mat beneath the subjects. 

### 2.3. QCT BMD Calculation from MDCT Data

BMD values for all lumbar vertebrae were calculated from the following relation: BMD_MDCT_ = [HAb/ (HUb − HUw)] × (HU − HUw) [29]. HUb and HUw represent the image intensity of bone and water-like phantoms. HAb = 200 mg/mL and HAw = 0 mg/mL are two phases in the Siemens Osteo Phantom, representing bone and water, respectively. Hounsfield units (HU) were calculated for each vertebra from L1 to L3 from regions of interest (ROIs), which were manually placed at equal distances from both the endplates in the trabecular components of the anterior vertebral body by a radiologist. Standard BMD _QCT_ for each vertebra was calculated from MDCT-related BMD using the following relation: BMD_QCT_ = 0.69 × BMD_MDCT_−11 mg/mL [30]. We averaged the L1 to L3 BMD values for the whole lumbar model, and the calculated BMD_QCT-L1-L3_ values were used as standard BMD_QCT_ values in the current study.

### 2.4. MDCT Image Segmentation and Lumbar Spine Modeling

Using a deep learning-driven framework (https://anduin.bonescreen.de; accessed on 5 January 2021) [31,32,33], the L1–L5 vertebrae were automatically labeled and segmented. This algorithm was semi-automated, accurately identifying the spine regions and creating separate segmentation masks for each vertebra [31,32,33]. 

The MDCT data and segmentation masks were then imported to the open-source 3D image reconstruction software 3D Slicer (version 4.11; https://www.slicer.org/; accessed on 20 July 2021) for patient-specific vertebral body generation. The segmented model was then imported to the commercial FE tool Abaqus CAE (version 6.10; Johnston, Rhode Island, United States) for meshing. To capture the irregular shape of the vertebrae accurately, we used linear tetrahedral elements (C3D4) for meshing. The meshed vertebral bodies were then imported to the open-source material mapping tool Bonemat (version 3.2; http://www.bonemat.org/; accessed on 15 August 2021). The vertebral models were then mapped with image intensity-specific material properties using this software program. The material mapping relations used in the current study are shown in Table 1. 

**Figure 2:**
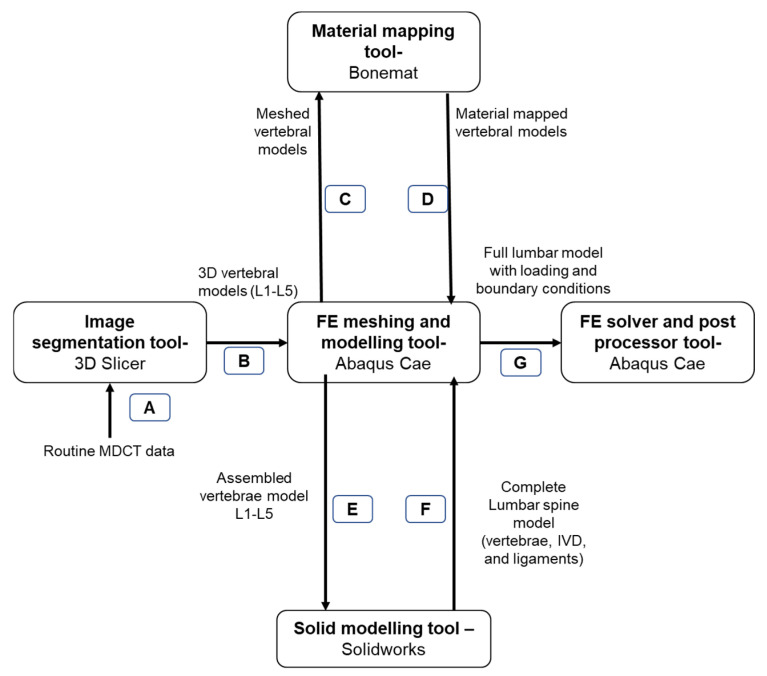
The flow of segmented models between different processing tools in the current computational study. A—Importing the routine clinical data to image segmentation tool; B—Importing of segmented vertebral models to finite element (FE) preprocessor; C—Importing of meshed vertebral models to material mapping tool; D—Importing of material-mapped models to FE preprocessor; E—Importing of the assembled vertebral model to solid modeling tool for the generation of IVD and ligaments; F—Importing the complete lumbar spine model to FE preprocessor; G—Application of loading and boundary conditions to the lumbar model and importing to FE solver.

The material-mapped vertebral models were then returned to Abaqus CAE software (version 6.10; Dassault Systems, Johnston, RI, USA). For IVD and ligament generation, the imported vertebrae were then assembled and exported to Solidworks (version 2021; Dassault Systems, Waltham, MA, USA). In this software, the nucleus pulposus and annulus fibrosus of the IVD and the ligaments were manually generated. The nucleus surface area was maintained at 30% of the overall IVD area [15,43]. The flow of the segmented models between different tools is shown in Figure 2. 

The ligaments were modeled manually as 3D wire elements based on the anatomical positions. In the current study, we have considered a total of seven ligaments, namely: anterior longitudinal ligament (ALL), posterior longitudinal ligament (PLL), interspinous ligament (ISL), supraspinous ligament (SSL), ligamentum flavum (LF), facet capsular ligament (FCL), and intertransverse ligament (ITL). The properties of the ligaments used in the current study are shown in Table 2. 

### 2.5. FE Modeling and Analysis

**Figure 3:**
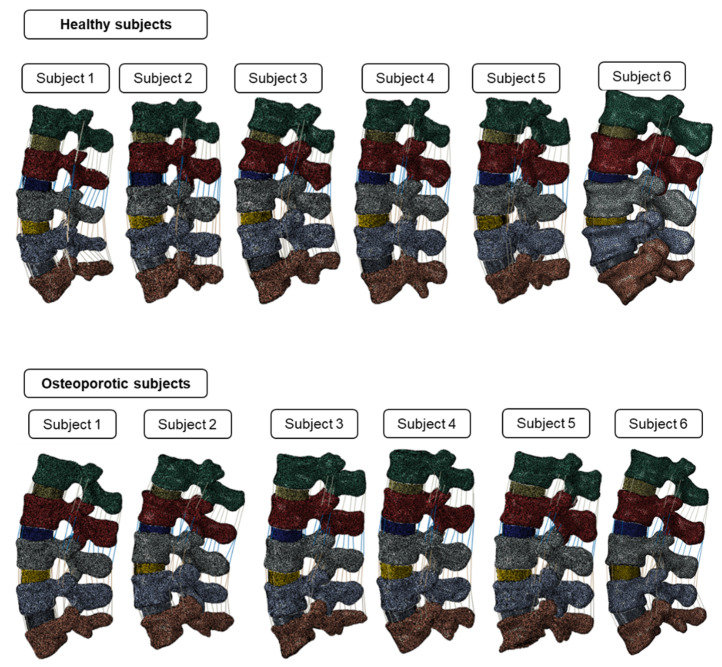
The meshed comprehensive lumbar spine geometries used in the current study. The top row shows the healthy subjects; the bottom row shows the osteoporotic subjects.

Modeled IVDs and ligaments were then imported to Abaqus CAE for further analysis. The vertebrae, IVDs, and ligaments were assembled, and a comprehensive patient-specific lumbar model was generated. The meshed geometry models used in the current study are shown in Figure 3. To maintain the accuracy of the computational model, we performed a mesh sensitivity analysis by varying the element edge length from 0.5 mm to 2.5 mm for vertebrae and 0.25 mm to 1.5 mm for IVDs with an increment of 0.25 mm. We observed that 1.5 mm and 0.75 mm element edge sizes produced a mesh-independent result for the vertebrae and IVDs, respectively. Thus, those element sizes have been used to mesh all the lumbar models.

**Figure 4:**
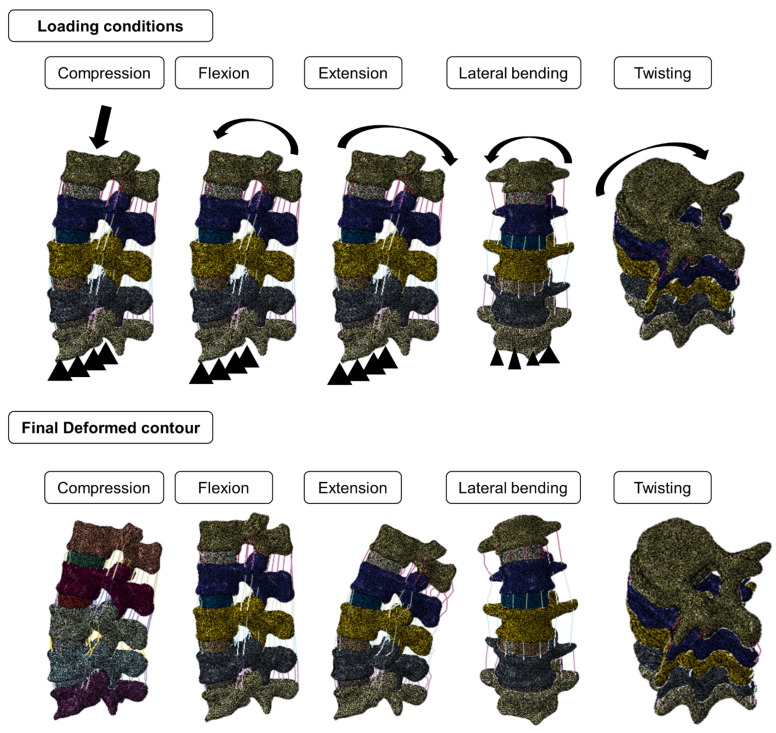
Applied loading and final deformed contours of the lumbar spine models. The top row shows the different loading conditions, i.e., compression, flexion, extension, lateral bending, and twisting. The bottom row shows the final deformation contours for the abovementioned loading conditions.

For replicating the realistic mechanical behavior of the model under the applied loading condition, a tie constraint was given between nucleus and annulus of IVDs, nucleus and vertebrae, annulus and vertebrae, and ligaments and vertebrae [45]. The inferior surface of the lumbar spine was fixed in all six directions. For calculation of the FL, normal displacement load was applied on the superior surface, the model was simulated, and the variation of load and displacement was captured. The peak of the load-displacement curve was considered the FL. This methodology was validated with experimental studies in previous work [15,20]. For studying the kinematics, we applied a pure moment of 7.5 Nm on the superior surface of the lumbar spine and simulated flexion, extension, lateral bending, and twisting motions [46]. In addition, we have plotted the variation of ROM with angular displacement. This methodology was validated in the previous works [21,42]. The applied loading and boundary conditions and the final deformed contour after the simulation are shown in Figure 4.

### 2.6. Statistical Data Analysis

Statistical analyses were performed using Microsoft Excel (version16.0; Redmond, WA, United States) and IBM SPSS Statistics for Windows (version 25.0; IBM Corp., Armonk, NY, USA). We used the descriptive statistics for FL and ROM parameters, calculated mean ± SD, and compared it with previously published experimental results. Correlation plots were plotted to observe the relation between FL and ROM parameters, and BMD_QCT-L1-3_ and Spearman correlation coefficients (ρ) were calculated. We performed Mann–Whitney U tests at a significance level of 0.05 to compare the FL and ROM parameters of HC and OP.

## 3. Results

### 3.1. Calculation of FL and ROM Values for Healthy and Osteoporotic Lumbar Models 

**Figure 5:**
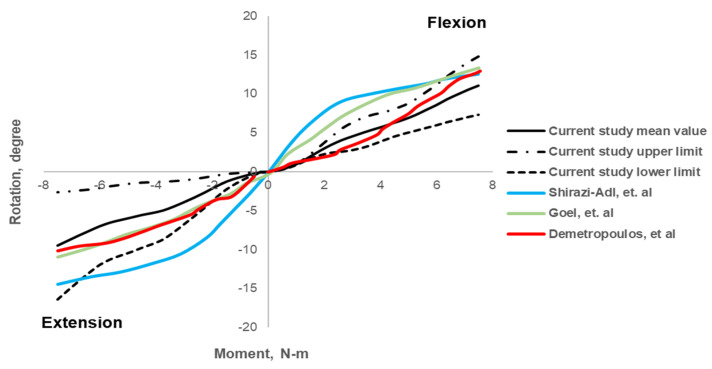
Variation of angle with respect to applied moment on the superior surface of the lumbar model during flexion and extension.

**Figure 6:**
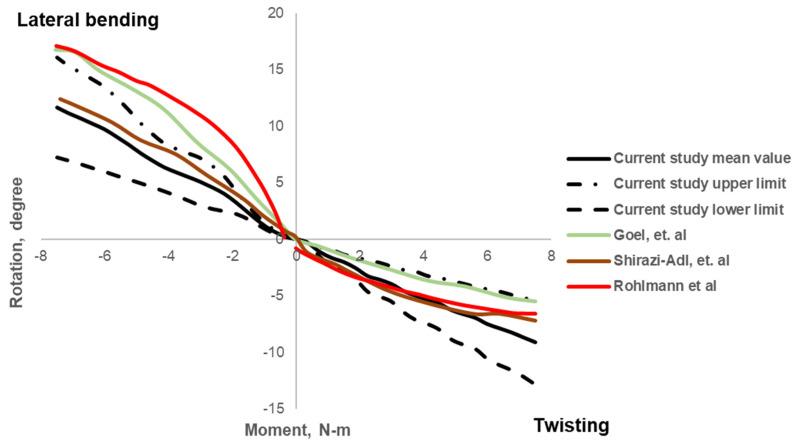
Variation of angle with respect to applied moment on the superior surface of the lumbar model during lateral bending and twisting movement.

The mean age of HC is 62.17 ± 10.24 years (range: 43 to 75 years), and for OP is 65.83 ± 11.19 years (range: 41 to 73 years). The BMD_QCT-L1-3_ and FE-predicted FL values for the healthy lumbar spine were 100.77 ± 9.25 mg/mL and 1471.50 ± 275.69 N, respectively. The maximum mean rotation values for the applied moment were as follows: 11.11° ± 3.73° for flexion (F), 12.05° ± 6.12° for extension (E), 11.80° ± 4.36° for lateral bending (L), and 8.96° ± 3.72° for twisting (T). The variations in rotation values with respect to the applied moment are shown in Figure 5 and Figure 6. The BMD_QCT-L1-3_ and FE-predicted FL values for the osteoporotic lumbar spine models were 61.21 ± 3.67 mg/mL and 763.33 ± 166.70 N, respectively. The maximum mean rotation values were as follows: 11.26° ± 2.02° (F), 14.75° ± 3.93° (E), 14.37° ± 3 (L), and 12.48° ± 2.25° (T).

**Figure 7:**
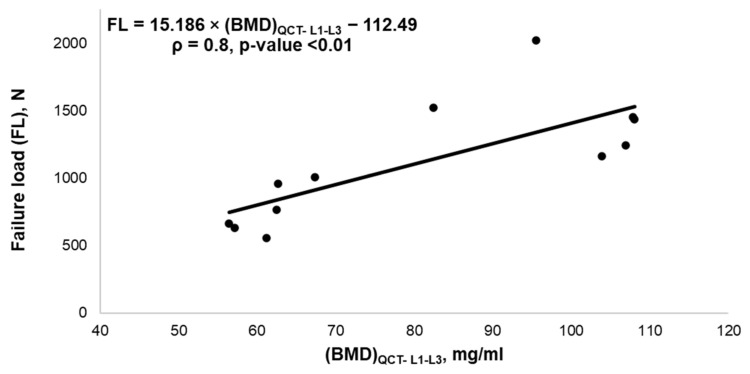
Correlation plot between FE-predicted failure load and QCT BMD, ρ—Pearson correlation coefficient.

**Figure 8:**
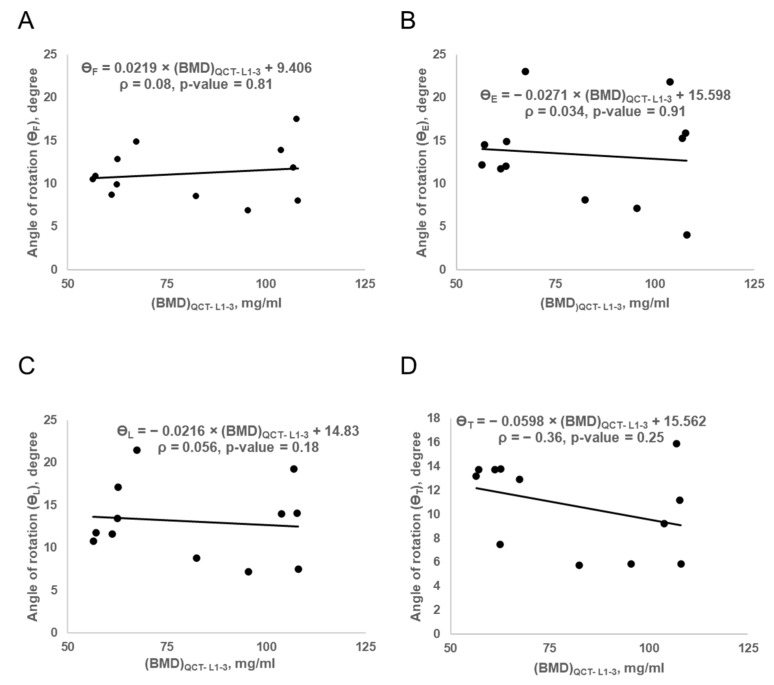
Correlation plot between ROM parameters and QCT BMD: (**A**)—flexion angle of rotation (ϴ_F_) vs. QCT BMD; (**B**)—extension angle of rotation (ϴ_E_) vs. QCT BMD; (**C**)—lateral bending angle (ϴ_L_) vs. QCT BMD; (**D**)—twisting angle of rotation (ϴ_T_) vs. QCT BMD, ρ—Spearman correlation coefficient.

We observed a strong correlation (R^2^: 0.8, *p* < 0.01) between FE-predicted FL and BMD_QCT-L1-L3_ values in all subjects. The correlation plot is shown in Figure 7. We observed no statistically significant correlations between the ROM parameters and BMD_QCT-L1-3_ values, ρ = 0.08, *p* = 0.81 (F); ρ = 0.034, *p* = 0.91 (E); ρ = 0.056, *p* = 0.18 (L); ρ = −0.36, *p* = 0.25 (T). The correlations between the ROM parameters and BMD values are shown in Figure 8.

### 3.2. Comparison of Healthy and Osteoporotic Lumbar Spine Models 

**Figure 9:**
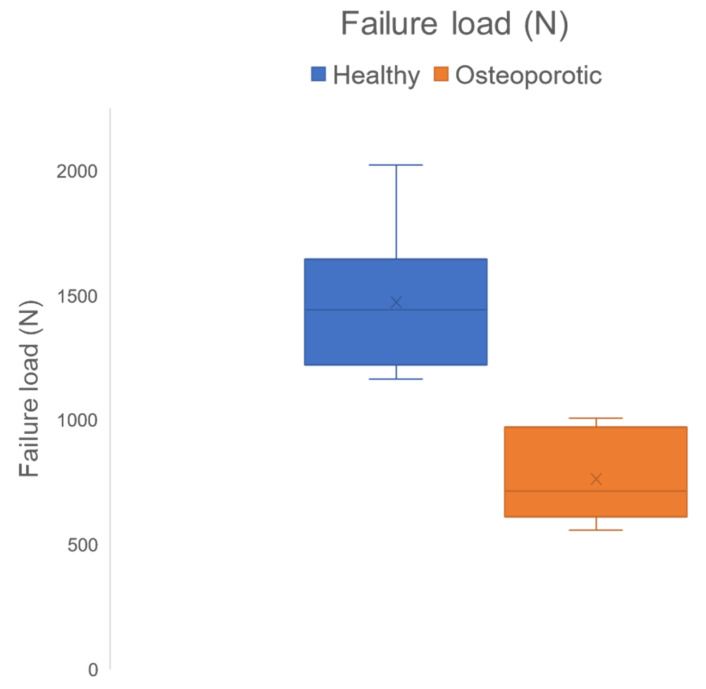
Box plot showing the distribution of failure load values for healthy and osteoporotic lumbar spine models.

We observed a significant difference between healthy and osteoporotic lumbar spine models for BMD_QCT-L1-3_ (*p* < 0.01) and FE-based FL values (*p* < 0.01). The FL values were higher for HC compared to those of OP (1471.50 ± 275.69 N vs. 763.33 ± 166.70 N). The ROM parameters were higher for OP compared to HC: 11.11° ± 3.73° vs. 11.26° ± 2.02° (F); 12.05° ± 6.12° vs. 14.75° ± 3.93° (E); 11.80° ± 4.36° vs. 14.75° ± 3.93° (L); 8.96° ± 3.72° vs. 12.48° ± 2.25° (T). Even though the mean rotation values for the osteoporotic models were higher than for the healthy models, no significant differences were observed between HC and OP for the ROM parameters: *p* = 0.69 (F), *p* = 0.69 (E), *p* = 0.47 (L), and *p* = 0.13 (T), respectively. The mean, SD, and *p*-values for different parameters for healthy and osteoporotic lumbar spine models are shown in Table 3. Figure 9 shows the FE-derived FL results as a box plot.

## 4. Discussion

The current work studied the feasibility of using clinical routine MDCT data to model the whole lumbar spine with a FE method to derive FL and ROM parameters. Our results showed that the FE-based FL values and ROM parameters for healthy lumbar models were in the range of previously published experimental and other computational studies. Furthermore, in contrast to ROM parameters, FL significantly differed between HC and OP. Thus, the routine clinical image data can potentially be used to model the lumbar spine. 

We have demonstrated the use of the clinical routine MDCT data for modeling and studying the whole lumbar spine behavior at different loading conditions using FE analysis. The spine has a complex configuration with continuous interaction between tissues and structures such as vertebrae, IVDs, and ligaments. Lower back pain (LBP) is one of the prevalent medical conditions, affecting around 90% of adults worldwide [47]. In 2015, around 540 million were suffering from activity-limiting back pain (around 7.3% of the global population). Untreated LBP can cause permanent disability y [48]. LBP can occur due to multiple reasons such as disk herniation [49,50], osteoporosis [51], weight [52], etc. Osteoporotic fractures were one of the major causes of LBP. As stated earlier, understanding in vivo lumbar spine biomechanics is essential to identifying, assessing, and predicting various pathophysiological conditions and monitoring clinical treatments. Due to the complex geometrical and topological characteristics (size and shape) and physiological loading conditions, direct in vivo measurement of the lumbar spine biomechanical aspects is challenging. In addition, vertebral compression fractures severely affect the spine biomechanics, which results in secondary fractures. The primary functions of the lumbar spine are the range of motion (ROM) to perform day-to-day activities, provide stability and maintain balance, and sustain the weight of the upper body. Thus, it is essential to analyze the lumbar spine as an anatomical and functional unit to understand its biomechanical behavior better.

Identifying the osteoporosis cases in advance is feasible using the FE-MDCT methodology. However, most FE-based studies have used high-resolution images (low slice thickness of 0.5 to 1.5 mm and no contrast agent) for bone analysis. Studies have shown that the radiation can reach up to 5.6 mSv for the conventional CT scans, which is relatively high compared to the DXA scan used to quantify the bone mineral density and diagnose osteoporosis. High radiation risk could potentially discourage using FE analysis even when we used routine clinical data to develop the model. So, it is vital to study the feasibility of using routine clinical data for FE analysis. The primary issue with MDCT images acquired during the clinical routine for diagnosis is their lower spatial resolution than those acquired in experimental settings, thus causing partial volume effects. Rayudu et al. showed that routine data could be used for osteoporotic fracture risk assessment based on a single vertebral failure load derived from FE analysis [26]. 

We have observed that FE-derived FL for the healthy lumbar spine models was 1471.50 ± 275.69 N. These FE-predicted failure load values are in a comparable range with the available previous experimental results of 967 N to 4387 N [53]. The flexion (F), extension (E), lateral bending (L), and twisting (T) were similar to those in previous experimental results and computational studies [46,53,54,55]. The maximum mean ROM values were also in the range of previously published literature: 11.11° ± 3.73° vs. 12.59° to 13.36° for F [53,55,56]; 12.05° ± 6.12° vs. 10.12° to 14.45 for E [46,55,56]; lateral bending of 11.80° ± 4.36° vs. 12.39° to 17.08° for L [46,55,56]; and twisting of 8.96° ± 3.7° vs. 5.46° to 7.16° for T [46,55,56]. Based on these results, we can conclude that the routine clinical data could be used to model and analyze the lumbar section of the spine and generate comparable results.

The FE-derived FL was significantly lower in OP (763.33 ± 166.70 N) compared to HC (1471.50 ± 275.69 N). The osteoporotic vertebrae were expectedly weaker due to reduced bone mass and deterioration of trabecular microstructure. Chandran et al. have shown significantly higher values in FE-derived FL for healthy models compared to osteoporotic for the femoral bone [57]. In addition, Hatira et al. observed that the strength of osteoporotic lumbar vertebrae was lower than that of healthy models [58]. The results of the present study also follow a similar pattern of difference. Specifically, BMD_QCT-L1-3_ and FE-derived FL were significantly higher in HC when compared to in OP subjects. This trend is in line with previously reported studies, which have shown that FE-derived FL and vBMD values could predict vertebral fractures more effectively with an area under the curve (AUC) = 0.804 and 0.815, respectively, compared to aBMD values (AUC = 0.715) [23].

Interestingly, although the mean ROM values for the osteoporotic models were higher than those of the healthy models, ROM parameters were not significantly different between HC and OP. Tsouknidas et al. have observed around 15% variation in the ROM parameters between osteoporotic models and healthy sacro-lumbar spine models [59]. In this regard, vertebrae weaken with aging and show increased porosity due to osteoporosis. Somovilla-Gómez et al. showed that the maximum F-angle variation is under 1° from age 35 to 64 years [60]. They have also developed a parametric model to explore the variation of ROM parameters with age. Using this model, when a 30-year-old man (weight = 70 kg, height = 160 cm) and 55-year-old man (weight = 70 kg, height = 160 mm) were compared, the differences in ROM were as follows: 0.15° (F), 0.477° (E), 0.11° (L), and 0.08° (T) [60]. From these values, we might conclude that osteoporosis seems to have a lower influence on ROM parameters than FE-derived FL values. In the present study, we have observed that osteoporosis severely affects bone strength and less affects lumbar spine ROM. 

The FE-based lumbar spine modeling methodology developed in this study can be used to study various downstream applications such as disk herniation and osteoporosis [49,50]. With opportunistic screening, it will be possible to predict different clinical conditions such as vertebral fractures, weekend disc, or so before herniation occurs. Furthermore, FE-based patient-specific models developed from opportunistically acquired MDCT data can be used to understand the changes in the spine biomechanics, in turn predicting those conditions. The complexity and risk involved with spine surgery necessitate the need for pre-operative visualization of anatomical and pathological structures and planning of the procedures to minimize the risk and improve the surgical outcome. 3D intraoperative optical systems offer clear visualizations to aid surgeons and have been proven to be effective. However, most of the planning of the procedure is being conducted with static medical images (MRI, CT, and radiographs) and intraoperatively guided via ultrasound and fluoroscopic imaging systems. Finite element simulations offer enriched information on tissue health and target anatomical locations during the planning phase of the surgical procedure. In addition, reconstructed anatomical models of the spine also provide 3D geometric and topological characteristics to visualize and plan effectively. 

There are some limitations in the current study that need to be considered while interpreting the results of this study. First, the cohort size was relatively small, thus restricting the statistical power. Second, we have considered linear elastics properties for the IVDs with annulus and nucleus due to computational resource limitations. We aim to incorporate more realistic IVD modeling in our future studies. Third, we have only simulated the lumbar model under static loading conditions. The results may vary when the model is simulated under dynamic conditions such as gait and other daily activities. Fourth, even though the age range of these cohorts is similar, the smaller sample size resulted in higher variability.

## 5. Conclusions

We investigated the feasibility of using clinical routine MDCT data to model the whole lumbar spine as the functional spine unit. We observed healthy correlations between FE-derived lumbar spine models and experimental results. In addition, we found significant differences in FL between healthy and osteoporotic subjects. ROM parameters were not significantly different between HC and OP. From these findings, we may conclude that the FE-based models developed from clinical routine MDCT data can be used for analyzing the whole lumbar spine. These FE-based models could be used as a complementary tool to the existing BMD-based measures in osteoporosis to assess the patient-specific status of the biomechanical properties of the whole lumbar spine.

## Figures and Tables

**Table 1:** Elastic constants and material mapping relations used in modeling vertebrae and IVDs in this study. The density values were calculated from Hounsfield units (HU), and then the modulus and stress values were derived from densities.

Mechanical Property	Mapping Relations
Vertebrae material properties	
Apparent density (ρ_app_ in Kg/m^3^) [34]	ρ_app_ = 47 + 1.122 × HU
Ash density (ρ_ash_ in Kg/m^3^) [35]	ρ_ash_ = 0.6 × ρ_app_
Elastic modulus (E in MPa) [34,36]	Ez = 4730 × (ρapp)^1.56^ Ex = Ey = 0.333 Ez Z- Vertebrae axial direction
Shear modulus (G in MPa) [37]	Gxy = 0.121 Ez Gxz = Gyz = 0.157 Ez
Poisson ratio (V) [37]	Vxy = 0.381 Vxz = Vyz = 0.104
Maximum principal stress limit (σ in MPa) [38]	σ = 137 × ρ_ash_ ^1.88^, ρ_ash_ < 0.317 σ = 114 × ρ_ash_ ^1.72^, ρ_ash_ > 0.317
Plastic strain (εAB) [39]	ε_AB_ = −0.00315 + 0.0728 ρ_ash_
Minimum principal stress limit (σ_min_ in MPa) [39]	σ_min_ = 65.1 × ρash ^1.93^
Intervertebral disc properties	
Annulus	
Elastic modulus (E in MPa) [40]	E = 25
Poisson ratio (V) [40]	0.49
Density (ton/mm^3^) [41]	1.20 × 10^−9^
Nucleus	
Elastic modulus (E in MPa) [42]	E = 1
Poisson ratio (V) [42]	0.49
Density (ton/mm^3^) [41]	1.00 × 10^−9^

**Table 2:** The mechanical material constants used for modeling the wire ligaments. Circular cross-sectional area and the ligament number [41,44].

	Density (Ton/mm^3^)	Youngs Modulus (MPa)	Poisson’s Ratio	Circular Cross-Sectional Area (mm^2^)	Number of Ligaments
ALL	1 × 10^−9^	55.77	0.4	32.4	3
PLL	1 × 10^−9^	54.43	0.4	05.2	3
LF	1 × 10^−9^	03.25	0.4	84.2	3
ISL	1 × 10^−9^	02.23	0.4	35.1	4
SSL	1 × 10^−9^	12.80	0.4	25.2	2
ITL	1 × 10^−9^	11.50	0.4	12.0	4
FCL	1 × 10^−9^	08.69	0.4	43.8	6

**Table 3:** Comparison of BMD, failure load, and ROM parameters for healthy and osteoporotic subjects and level of significance values. The asterisks (*) indicate statistical significance (*p* < 0.01).

	Healthy		Osteoporotic		*p*-Value
	Mean	Std Dev	Mean	Std Dev	
QCT BMD (mg/mL)	100.77	9.25	61.21	3.67	<0.01 *
Failure load (*n*)	1471.50	275.69	763.33	166.70	<0.01 *
Flexion (°)	11.11	3.73	11.26	2.02	0.69
Extension (°)	12.05	6.12	14.75	3.93	0.69
Lateral bending (°)	11.80	4.36	14.37	3.78	0.47
Twisting (°)	8.96	3.72	12.48	2.25	0.13

## Data Availability

The raw data supporting the conclusions of this article will be made available by the authors without undue reservation.

## Abbreviations

3D	Three-dimensional
aBMD	Areal bone mineral density
vBMD	Volumetric bone mineral density
QCT	Quantitative computed tomography
CT	Computed tomography
DXA	Dual-energy X-ray absorptiometry
QCT	Quantitative computed tomography
HU	Hounsfield units
MDCT	Multi-detector computed tomography
FE	Finite element
PACS	Picture archiving and communication system
SD	Standard deviation
ROC	Receiver operating characteristic
AUC	Area under the curve
BMD_QCT-L1-L3_	Vertebrae
FL	Failure load
F	Flexion loading
E	Extension loading
L	Lateral bending loading
T	Twisting loading
ρ	Spearman coefficient
WHO	World Health Organization
HC	Healthy controls
OP	Osteoporotic patients
FRAX	Fracture Risk Assessment Tool
ALL	Anterior longitudinal ligament
PLL	Posterior longitudinal ligament
ISL	Interspinous ligament (ISL)
SSL	Supraspinous ligament
LF	Ligamentum flavum
FCL	Facet capsular ligament
ITL	Intertransverse ligament
IVD	Intervertebral disk
